# Older Adults’ Current and Maximum Acceptable Travel Times for Primary Care: Results From a National Survey

**DOI:** 10.1093/geroni/igaf122.2014

**Published:** 2025-12-31

**Authors:** Tabasa Ozawa, Ying Liu, Soeren Mattke

**Affiliations:** University of Southern California, Los Angeles, California, United States; University of Southern California, Los Angeles, California, United States; University of Southern California, Los Angeles, California, United States

## Abstract

Long travel times may create obstacles to care, particularly for older adults who may need frequent care and assistance with transportation. However, little data exists on how much travel time might prevent care-seeking. We surveyed 5,453 individuals aged 50+ in the nationally representative Understanding America Study about their willingness to travel for primary care and examined the factors associated with preference. Most respondents (80%) currently traveled 0-30 minutes for primary care, 14% 31-60 minutes, 2% more than 60 minutes, with 3% having no primary care provider. The vast majority (94%) traveled by car. Asked about the maximum travel time before delaying or forgoing care, 28% responded 0-30 minutes, 42% 31-60 minutes, and 30% more than 60 minutes. Most (77%) were willing to travel longer than their current time. Logistic regression showed older age, higher income, belonging to non-Hispanic white group, higher education, poorer self-rated health, and shorter current travel time were significantly associated with greater willingness to travel longer than current. Income and race/ethnicity had strong effects: 83% of individuals with income ≥$100,000 were willing to travel longer, versus 69% of those with income ≤$39,000 (odds ratio [OR]=2.41, p < 0.0005); 81% for non-Hispanic White versus 67-68% for racial/ethnic minority groups (OR = 2.14-2.22, p < 0.0005). The findings suggest that most older adults would be accepting of longer travel times rather than forgoing care, but there are notable differences by demographic groups. Further research should investigate whether these stated preferences correspond to actual behavior, and whether and how much travel times limit access.

